# Impact on Respiratory Mechanics of Uniportal–Biportal Robotic Lung Resection Without Capnothorax Versus Multiportal Approach With Capnothorax: A Prospective Cohort Observational Study

**DOI:** 10.1155/anrp/5324693

**Published:** 2026-10-08

**Authors:** Ricard Navarro-Ripoll, Stefano Italiano, Ferran Seguí, Álvaro Buñuel, David Revuelta, Manuel López-Baamonde, Anna Ureña, Ricard Ramos, Carlos Ferrando

**Affiliations:** ^1^ Department of Anesthesia and Critical Care Clinic Hospital Barcelona Spain; ^2^ Institut D’Investigacions Biomèdiques August Pi I Sunyer (IDIBAPS) Barcelona Spain idibaps.org; ^3^ Department of Surgery and Medical Specializations Faculty of Medicine and Health Sciences University of Barcelona (UB) Barcelona Spain ub.edu; ^4^ Department of Anesthesia and Critical Care Hospital Universitari de Bellvitge Hospitalet de Llobregat Spain bellvitgehospital.cat; ^5^ Department of Thoracic Surgery Clinic Hospital Barcelona Spain; ^6^ CIBER de Enfermedades Respiratorias Instituto de Salud Carlos III Madrid Spain isciii.es

**Keywords:** capnothorax, mechanical power, respiratory mechanics, robotic-assisted thoracic surgery (RATS)

## Abstract

**Background:**

Several robotic‐assisted thoracic surgery (RATS) techniques have been described. Main differences among them are the location of ports and CO_2_ insufflation, with a potential impact on respiratory mechanics. This study assesses the respiratory impact of uniportal or biportal RATS (uRATS) without capnothorax versus a historical cohort of patients who had multiportal RATS (mRATS) with capnothorax.

**Methods:**

Prospective, observational, and descriptive study enrolling adult patients (> 18 years/o) undergoing elective uRATS lung resection and comparison to an historical cohort of 30 mRATS patients, from July 2023 to February 2024. Protocolized recruitment maneuvers (RMs) were performed, and optimal positive end–expiratory pressure (PEEP) to best compliance was calculated during one‐lung ventilation (OLV). Respiratory parameters were collected intraoperatively: during two‐lung ventilation (TLV, T1), OLV before the RM (T2), OLV after the RM (T3), 20 min after the RM (T4), at the end of OLV (T5), and before extubation (T6). To test quantitative variables, a linear mixed‐effects model was used.

**Results:**

A total of 30 patients undergoing uRATS were enrolled and compared to 30 patients undergoing mRATS in a previous study. At T3, patients in the uRATS group needed significantly lower PEEP (mean difference 5.3 cmH_2_O; *p* < 0.001), which was also translated to a lower *P*_plat_ (4.2 cmH_2_O; *p* < 0.001) and *P*_peak_ (4 cmH_2_O; *p* = 0.003). These differences were maintained at T4 and T5 but not at T6. Patients in the uRATS groups showed lower mechanical power at T4 and T5 (mean difference at T4 4.8 J/min, *p* < 0.001, and at T5 5.0 J/min, *p* < 0.001).

**Conclusions:**

Uniportal and biportal robotic lung resection without capnothorax is associated with a lower impact on respiratory mechanics when compared to the multiportal robotic approach with capnothorax.

**Trial Registration:** ClinicalTrials.gov identifier: NCT06784037

## 1. Introduction

Robotic‐assisted thoracic surgery (RATS) is being widely implemented thanks to its potential benefits, including lower postoperative complications, length of stay, and enhanced recovery [1]. However, postoperative pulmonary complications (PPCs) remain the most common complication after robotic lung resection [2]. Intraoperative strategies designed to decrease PPC, such as low tidal volume (VT) ventilation, use of recruitment maneuvers, and positive end–expiratory pressure (PEEP) titration may influence its incidence and thus the postoperative outcome [3].

Several robotic techniques have been described [4]. Main differences among them are the number and location of ports and/or assistant access, use of minithoracotomy, number of robotic arms used, and CO_2_ insufflation. Uniportal approach reproduces the uniportal thoracoscopic surgery frontal view, adding 3D vision, enhanced precision, and wristed instrument articulation. Hilar and mediastinal dissection follow the same principles as in thoracoscopic surgery, but limited working space requires precision, coordination, and significant experience [5]. Biportal approach provides greater separation of instruments and facilitates triangulation, thereby reducing collisions and improving surgical ergonomics [6]. Finally, during multiportal RATS (mRATS), tension capnothorax is mainly used to improve the surgical field, facilitating lung collapse and creating a mediastinal and diaphragmatic shift, thus helping tissue resection [7].

Tension capnothorax is associated with increased respiratory driving pressure (DP), higher PEEP requirement, and significant hypercapnia [8]. Robotic approaches without capnothorax may avoid CO_2_ detrimental effects while preserving the benefits of robotic surgery. However, to the best of our knowledge, there are no comparative studies assessing the intraoperative impact on respiratory mechanics of different robotic approaches. We designed a prospective cohort study to assess the impact on respiratory mechanics and gas exchange of uniportal and biportal (uRATS) without tension capnothorax versus a historical cohort of patients who had a multiportal (mRATS) robotic lung resection using tension capnothorax.

## 2. Materials and Methods

### 2.1. Study Design and Participants

This prospective observational cohort study was performed at Hospital Clínic’s Department of Anesthesiology and Critical Care, Barcelona, Spain. It was designed in compliance with the fundamental principles established in the Declaration of Helsinki and the Convention of the European Council related to human rights and biomedicine. The final protocol was approved by the Hospital Clinic Ethical Committee (HCB/2024/0103), and written informed consent was obtained from all patients. The manuscript adheres to the Strengthening the Reporting of Observational Studies in Epidemiology (STROBE) guidelines [9].

Adult patients (> 18 years/o) undergoing elective uniportal or biportal RATS lung resection with a da Vinci platform (Intuitive Surgical Inc., Sunnyvale, CA, USA) who agreed to participate in the study and signed the written consent form were included. Exclusion criteria were (1) ASA physical status IV; (2) pneumonectomy; (3) New York Heart Association > III; and (4) preoperative hemoglobin < 10 mg/dL, and patients in whom recruitment maneuvers were contraindicated.

### 2.2. General Procedures

Patients received routine monitoring, including electrocardiogram, pulse oximetry, invasive arterial blood pressure, neuromuscular monitoring (train‐of‐four, TOF‐Cuff, RGB Medical Devices SA), depth of anesthesia (Bispectral Index), and esophageal temperature. Induction and maintenance of general intravenous anesthesia were performed with propofol, remifentanil, and rocuronium. An appropriately sized left‐side double lumen tube (Mallinckrodt) was used for tracheal intubation. The tube position was confirmed by bronchoscopy in the supine and lateral positions. Lung ventilation was performed with Dräger Perseus A500 anesthesia machine. Patients were placed in lateral decubitus in the Pillet position, with flexion at the waist to widen intercostal spaces. Anti‐Trendelenburg was applied to prevent collision of the robotic arms with the hip.

### 2.3. Surgical Approach

Robotic thoracic surgeries were performed using three approaches: uniportal, biportal, and multiportal. As uniportal and biportal approaches were similar in the number and size of incisions and unrequired capnothorax, they were analyzed as a single group (uRATS). In contrast, the multiportal approach involved a greater number of incisions and the use of insufflation to facilitate surgical exposure. The uniportal robotic approach was performed through a single incision of approximately 3‐4 cm, usually located in the sixth or seventh intercostal space between the anterior and posterior axillary lines. Through this incision, the 30° camera and two robotic arms were introduced in parallel. The biportal robotic approach was based on the same incision described for the uniportal access, plus an additional incision placed two intercostal spaces below and in a more posterior position. Through the main incision, the 30° camera and one robotic instrument were introduced at the posterosuperior angle. Through the inferoposterior incision, a 12‐mm trocar was placed for the introduction of another robotic instrument.

The multiportal robotic approach was performed through 4‐5 ports placed along the seventh‐eighth intercostal space. CO_2_ insufflation at 5 mmHg was applied to facilitate lung collapse and increase the working space. An anterior 12‐mm trocar was inserted for the stapler, while the remaining ports were 8‐mm trocars for the robotic instruments and the camera. A schematic figure (Figure S1) in the supporting information provides a representation of the different incisions in every technique.

### 2.4. Study Variables

Ventilatory parameters monitored by the anesthesia machine included: VT, respiratory rate (RR), PEEP, fraction of inspiratory oxygen (FiO_2_), peak airway pressure (*P*_peak_), plateau pressure (*P*_plat_) calculated during 10% pause of inspiratory time, DP calculated as *P*_plat__t_ – PEEP, and quasistatic respiratory system compliance (*C*_rs_) calculated as VT/DP. Mechanical power (MP) was calculated as previously described as follows [10]:

MP (J/min) = 0.098 × VT × RR × (PEEP + 0.5 × [*P*_plat_−PEEP] + [*P*_peak_‐*P*_plat_]), and it was analyzed separately according to the different components: static (PEEP), dynamic elastic (*P*_plat_‐PEEP), and dynamic resistive (*P*_peak_‐*P*_plat_).

Other reported study variables were intraoperative hypoxemia, defined as PaO_2_/FiO_2_ < 60 mmHg, and hypercapnia defined as PaCO_2_ > 45 mmHg. Except for blood gases, all study variables were collected at 6 timepoints during surgery (Figure S2 in Supporting Appendix).

PPCs registered during the first seven postoperative days were respiratory failure, respiratory infection, pleural effusion, atelectasis, pneumothorax, bronchospasm, and acute respiratory distress syndrome. PPCs followed standard definition criteria [11].

### 2.5. Experimental Protocol

Ventilatory management was identical in both cohorts (Figure S2).

At T1 = Baseline two‐lung ventilation (TLV) (10 min after induction) volume control ventilation (VCV) was set with VT of 8 mL/kg of predicted body weight (PBW), 5 cmH_2_O PEEP, and 0.8 FiO_2_. The inspiratory‐to‐expiratory (I:E) ratio was 1:2 with an end‐inspiratory pause of 10%, and RR was adjusted to maintain the partial pressure of arterial carbon dioxide (PaCO_2_) between 35 and 45 mmHg.; T2 = Baseline one‐lung ventilation (OLV) (5 min after OLV initiation). Identical settings as described in T1, but a VT of 5‐6 mL/kg [12, 13]; T3 = OLV‐iPEEP (during OLV, right after the iPEEP was set as described below). Alveolar RM followed by a decremental PEEP titration trial was performed, as previously described [14]. The alveolar RM consisted of switching to pressure‐controlled ventilation (PCV) adjusting 20 cmH_2_O of DP, an inspiratory‐to‐expiratory ratio of 1:1, a RR of 15 breaths per minute, and a 5 cmH_2_O stepwise increase in PEEP every 5 respiratory cycles from 5 to 20 cmH_2_O of PEEP, achieving an airway opening pressure of 40 cmH_2_O. Thereafter, the iPEEP titration trial was initiated as follows. The ventilatory mode was switched back to VCV, adjusting the same baseline variables described except for PEEP, which was maintained at 20 cmH_2_O. Then, a 2 cmH_2_O stepwise decrease in PEEP every 15 s was done until the PEEP with the best C_rs_ (iPEEP) was obtained. Then, a new RM was performed, and patients were ventilated in VCV as described in T2 with iPEEP; *T*4 = 20′‐OLV‐iPEEP (20 min after T3 with identical surgical conditions). Ventilatory parameters did not change; T5 = End‐OLV‐iPEEP (at the end of OLV). Ventilatory settings did not change; T6 = End‐TLV (just before extubation, on TLV). Ventilatory settings as described in T1 with a new iPEEP. To calculate iPEEP, a new RM and iPEEP trial were performed as described in T3. Arterial blood gases were analyzed at T1, T4, T5, and T6, and 1 hour after tracheal extubation at the postanesthesia care unit (PACU) on oxygen nasal cannula at a flow of 2 L per minute.

After the PEEP trial, clinicians were allowed to modify the RR to a maximum of 20 breaths per minute and the I:E ratio to avoid intrinsic PEEP.

Arterial hypotension, defined as median blood pressure < 60 mmHg during RM and PEEP titration trial, was treated with phenylephrine boluses or noradrenaline infusion based on standard protocols. Total dosages of noradrenaline and phenylephrine were recorded.

The main difference between the uRATS cohort and the historical cohort of 30 patients’ mRATS from July 2023 to February 2024 is that in the mRATS patients, a continuous flow of CO_2_ with a pressure of 5 mmHg and a 5 L/min flow rate was given through a surgical port introduced in the thorax from T2 to T5. The capnothorax working pressure and flow remained unchanged during surgery. This decision was taken according to the surgical team previous experience and was maintained in all patients to simplify interpretation of changes on respiratory mechanics.

All study variables were collected at the same timepoints during surgery. The robotic platform used and perioperative care pathways were maintained throughout both study periods, including thoracic surgery and anesthesiology professionals.

### 2.6. Sample Size and Statistical Analysis

Based on the results of the previous mRATS cohort (mean PEEP at T3: 12.1 ± 2.1 cmH_2_O), a minimal clinically relevant difference of 2 cmH_2_O with a common SD of 2.5 would have required 26 patients per group (two‐sided *α* = 0.05, power = 80%). To ensure robustness, justify the planned recruitment and match the earlier cohort; the sample size for the URATS group was increased to 30. Data were presented as mean ± standard deviation (SD) or as median and interquartile range (IQR), depending on data distribution. Comparisons between groups were performed using Student′s *t*‐test or Mann–Whitney *U* test as appropriate. Categorical variables were compared using Pearson’s chi‐square test. A two‐tailed *p* < 0.05 was considered statistically significant. Normal distribution of each variable was assessed within each study group and at every time point using the Shapiro–Wilk test.

Longitudinal changes and between‐group differences over time were analyzed using a linear mixed‐effects model (LMM), which accounted for repeated measures by including a random intercept for each subject. Fixed effects were group, time, and their interaction (group × time). Model assumptions were checked graphically: residual‐versus‐fitted plots and groupwise boxplots were used to assess homoscedasticity, while Q–Q plots of residuals were used to assess their normality. No relevant deviations (from these assumptions) were observed, supporting the adequacy of the LMM approach. When a significant group × time interaction was identified, post hoc pairwise comparisons were performed using the Bonferroni correction to control for multiple testing. Data are expressed as mean difference and 95% confidence interval (CI). The adjusted *p* values reported were automatically computed by Stata in the post hoc analysis. Analyses of potential confounding factors are described in the Supporting Information. All analyses were performed using Stata 15 (StataCorp, College Station, TX, USA).

## 3. Results

From February 2024 to May 2025, 30 patients undergoing uRATS (9 uniportal and 21 biportal) were enrolled in this study. Demographic data are shown in Table 1 and compared to a previous cohort of mRATS from our hospital. There were no differences between groups in preoperative lung function test, Assess Respiratory Risk in Surgical Patients in Cataluña (ARISCAT) score, or basal pulse‐oximetry. The only difference between groups was the duration of OLV and duration of mechanical ventilation, which was lower in the uRATS group. Although this variable differed between groups, it was not included in the primary model because it represents a postexposure variable. A sensitivity analysis including OLV duration as a covariate is presented in the Supporting Appendix. Adjustment for OLV duration did not alter the findings of the primary analysis, with the overall pattern and statistical significance of group, time, and group‐by‐time effects remaining unchanged across all outcomes (Figure 1).

**TABLE 1:** Demographic data comparing mRATS and uRATS groups.

	mRATS (*n* = 30)	uRATS (*n* = 30)	*p*
Sex (Male/Female)	20 (66.6%)/10 (33.3%)	14 (46.7%)/16 (53.3%)	0.292^∗^
Age (years)	69 (9)	70.5 (12)	0.549^∗∗^
BMI (Kg/m^2^)	23 (3.6)	25.3 (4.7)	0.304^∗∗^
PBW (Kg)	63.4 (±8.1))	61.5 (±9.4)	0.424^∗∗∗^
FVC (%)	93.4 (±14.6)	102 0.2 (±17.5)	**0.049** ^∗∗∗^
FEV1 (%)	90 (23)	90 (19)	0.499^∗∗^
FEV1/FVC (%)	74.3 (8.4)	72.9 (10.7)	0.419^∗∗^
ARISCAT	50 (5)	50 (0)	0.359^∗∗^
Surgical side (L/R)	9 (30%)/21 (70%)	13 (43%)/17 (57%)	0.284^∗^
OLV duration (min)	163 (42)	113.5 (50)	**< 0.001** ^∗∗^
TLV duration (min)	39 (27)	46.5 (18)	0.089^∗∗^
MV duration (min)	200 (46)	167 (53)	**0.020** ^∗∗^

*Note:* Data are presented as median and IQR or mean ± standard deviation. Bold values indicate *p* < 0.05.

Abbreviations: BMI, body mass index; FEV1, forced expiratory volume in the first second; FVC, forced vital capacity; MV, mechanical ventilation; OLV, one‐lung ventilation; PBW, predicted body weight; TLV, two‐lung ventilation.

^∗^Chi‐square test.

^∗∗^Wilcoxon (Mann–Whitney) rank‐sum test.

^∗∗∗^Student’s *t*‐test.

**FIGURE 1:**
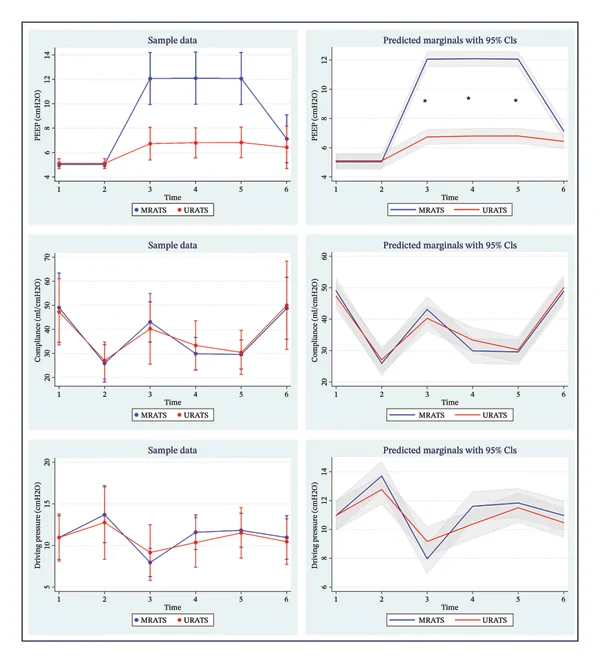
Comparison of respiratory mechanics between mRATS and uRATS. Sample data and predicted marginals by linear mixed‐effects model with 95% confidence interval. ^∗^ *p* < 0.05 using adjusted Bonferroni correction.

The overall fixed‐effect tests from the LMMs are presented in Table 2. All variables showed a significant effect of time (all *p* < 0.001). PEEP, plateau pressure, MP, static MP, RR and EtCO_2_ also demonstrated significant group‐by‐time interactions (all *p* < 0.001), whereas no overall group effect was observed for any variable. In contrast, neither DP nor respiratory system compliance showed a significant group‐by‐time interaction, indicating similar temporal patterns in both groups. Bonferroni‐adjusted pairwise comparisons are reported in Supporting Table S4.

**TABLE 2:** Overall fixed‐effect tests from the linear mixed‐effects models.

	Group *χ* ^2^	*p* value	Time *χ* ^2^	*p* value	Group × time *χ* ^2^	*p* value
PEEP	0.03	0.858	1693.76	< 0.001	527.41	< 0.001
Plateau pressure	0.01	0.937	294.06	< 0.001	112.92	< 0.001
Driving pressure	0.00	1.000	102.95	< 0.001	10.83	0.055
Compliance	0.36	0.550	235.69	< 0.001	5.50	0.358
Mechanical power	0.02	0.895	264.96	< 0.001	95.37	< 0.001
Static mechanical power	0.11	0.741	846.32	< 0.001	321.47	< 0.001
Respiratory rate	0.27	0.605	217.11	< 0.001	34.49	< 0.001
EtCO_2_	0.09	0.770	341.66	< 0.001	49.68	< 0.001

*Note:* Overall fixed‐effect tests derived from linear mixed‐effects models including group, time, and the group‐by‐time interaction as fixed effects, with patient included as a random intercept. Wald *χ* ^2^ are reported. PEEP: positive end–expiratory pressure. EtCO_2_: end tidal carbon dioxide.

No between‐group differences were observed at T1 or T2. At T3 (OLV after RM and individualized PEEP), patients in the uRATS group needed significantly lower PEEP (mean difference 5.3 cmH_2_O; 95% CI: 4.1–6.3; *p* < 0.001), which was also translated to a lower P_plat_ (4.2 cmH_2_O; 95% CI 1.4–7, *p* < 0.001) and P_peak_ (4 cmH_2_O; 95% CI: 0.7–7.4, *p* = 0.003). These differences were maintained at T4 at T5 but not at T6 (see Table 2, Figure 2, and Table S4 in Supporting appendix).

**FIGURE 2:**
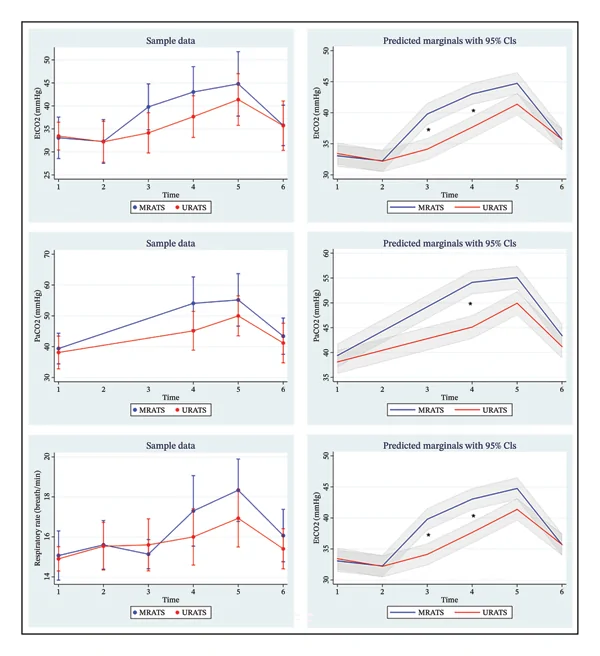
Comparison of EtCO_2_, PaCO_2_, and respiratory rate between mRATS and uRATS. Sample data and predicted marginals by linear mixed‐effects model with 95% confidence interval. ^∗^ *p* < 0.05 using adjusted Bonferroni correction. EtCO_2_: end tidal carbon dioxide; PaCO_2_: arterial partial pressure of carbon dioxide.

Patients in the uRATS groups showed lower MP at T4 and T5 (mean difference at T4 4.80 J/min, CI: 2.41–7.19 J/min, *p* < 0.001 and at T5 4.98 J/min, CI: 2.58–7.38, J/min, *p* < 0.001). When comparing different components, static MP was the only one that showed statistically significant differences between groups, seen at T3, T4, and T5 (see Table 3, Figure 3, and Table S3).

**TABLE 3:** Respiratory mechanics at different study times.

		T1	T2	T3	T4	T5	T6
PEEP (cmH_2_O)	mRATS	5.0 ± 0.2	5.0 ± 0.2	12.1 ± 2.1	12.1 ± 2.1	12.1 ± 2.1	7.1 ± 2.0
	uRATS	5.1 ± 0.4	5.1 ± 0.4	6.7 ± 1.3	6.8 ± 1.2	6.8 ± 1.3	6.4 ± 1.7

Plateau pressure (cmH_2_O)	mRATS	16.0 ± 2.7	18.7 ± 3.3	20.0 ± 2.7	23.7 ± 3.1	23.9 ± 3.0	18.1 ± 2.6
	uRATS	16.1 ± 2.9	18.0 ± 4.4	15.8 ± 4.2	17.2 ± 3.7	18.3 ± 3.5	16.9 ± 3.1

Driving pressure (cmH_2_O)	mRATS	11.0 ± 2.6	13.7 ± 3.4	8.0 ± 1.7	11.6 ± 2.1	11.8 ± 2.0	11.0 ± 2.6
	uRATS	11.0 ± 2.8	12.8 ± 4.4	9.2 ± 3.3	10.4 ± 3.0	11.5 ± 3.0	10.5 ± 2.7

Compliance (ml/cmH_2_O)	mRATS	49.0 ± 14.4	25.9 ± 7.8	43.1 ± 8.4	29.9 ± 6.7	29.6 ± 6.1	48.8 ± 12.8
	uRATS	47.3 ± 13.8	27.1 ± 7.7	40.3 ± 14.7	33.3 ± 10.3	30.5 ± 9.2	50.0 ± 18.3

Mechanical power (J/min)	mRATS	10.8 ± 2.4	8.1 ± 2.0	9.7 ± 2.5	13.0 ± 2.8	13.8 ± 3.0	13.4 ± 3.0
	uRATS	9.8 ± 2.7	7.8 ± 3.1	7.6 ± 3.1	8.2 ± 2.8	8.9 ± 2.6	11.0 ± 3.2

Static mechanical power (J/min)	mRATS	3.8 ± 0.6	2.6 ± 0.4	6.0 ± 1.4	6.9 ± 1.5	7.4 ± 1.7	5.7 ± 1.9
	uRATS	3.7 ± 0.8	2.6 ± 0.7	3.4 ± 1.2	3.5 ± 1.2	3.8 ± 1.2	4.7 ± 1.7

Respiratory rate (breath/min)	mRATS	15.1 ± 1.2	15.6 ± 1.2	15.1 ± 0.7	17.3 ± 1.8	18.3 ± 1.6	16.1 ± 1.3
	uRATS	14.9 ± 0.6	15.5 ± 1.2	15.6 ± 1.3	16.0 ± 1.4	16.9 ± 1.4	15.4 ± 1.0

EtCO_2_ (mmHg)	mRATS	33.1 ± 4.5	32.3 ± 4.8	39.8 ± 5.0	43.0 ± 5.5	44.8 ± 7.0	35.8 ± 4.4
	uRATS	33.4 ± 3.0	32.2 ± 4.4	34.1 ± 4.4	37.7 ± 4.5	41.4 ± 5.6	35.7 ± 5.4

*Note:* Data are presented as mean ± standard deviation.

Abbreviations: EtCO_2_, end tidal carbon dioxide; PEEP, positive end–expiratory pressure.

**FIGURE 3:**
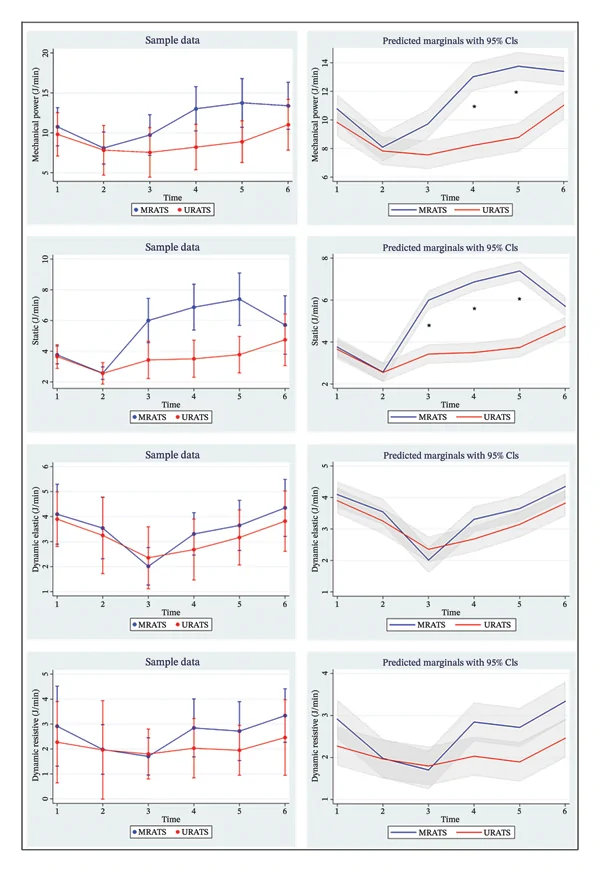
Comparison of mechanical power and its components (static, dynamic elastic, and dynamic resistive) between mRATS and uRATS. Sample data and predicted marginals by linear mixed‐effects model with 95% confidence interval. ^∗^ *p* < 0.05 using adjusted Bonferroni correction.

Patients in the uRATS group presented lower EtCO_2_ at T3 (mean difference 5.67 mmHg; CI: 1.44–9.90; *p* < 0.001). This difference was maintained at T4 (mean difference 5.37 mmHg; CI: 1.14–9.60; *p* < 0.01), when a lower PaCO_2_ (mean difference 9 mmHg; CI: 3.70–14.29; *p* < 0.001) and lower RR (mean difference 1.30 breath/min; CI: 0.21–2.39; *p* < 0.001) were also reported in the uRATS group. At T5, only the difference in RR was significant (mean difference 1.41 breath/min; CI: 0.32–2.51; *p* < 0.001) between groups. Patients in the uRATS group needed less total amount of vasopressors: median/IQR of total amount of noradrenaline in the uRATS 136 μg (372) vs. 352 μg (400) in the mRATS group (*p* = 0.006).

One of the 30 uRATS patients (3.33%) presented a chylothorax, and one patient (3.33%) developed a respiratory infection on postoperative Day 5.

## 4. Discussion

Our study has shown that uniportal and biportal (uRATS) robotic lung resection without capnothorax is associated with lower respiratory mechanical impact and less hypercapnia when compared to multiportal robotic approach using capnothorax (mRATS). Our study is of special interest because, to our knowledge, no previous study has compared the respiratory impact of different RATS techniques with or without capnothorax (Table 4).

**TABLE 4:** Comparison of noradrenaline total dose between mRATS and uRATS.

	MRATS (*n* = 30)	URATS (*n* = 30)	*p* value
Noradrenaline total dose (μg)	352 (400)	136 (372)	**0.006** ^∗^

*Note:* Data are presented as median and IQR. The bold value indicates *p* < 0.05.

^∗^Wilcoxon (Mann–Whitney) rank‐sum test.

Main objectives during OLV are to ensure oxygenation and ventilation without damaging the lung. Protective mechanical ventilation strategies include low VT, adequate PEEP, low DP, use of alveolar recruitment maneuvers, and lower MP [15]. When an individualized PEEP was applied, both groups from our study presented similar respiratory system compliance and DP. However, we have found that uRATS patients required less PEEP and thus it translated to lower plateau and peak pressures. The observed mechanical differences may reflect combined effects of surgical approach and capnothorax.

MP has proved to be useful when assessing patients with acute respiratory distress syndrome [16] and is associated with a greater risk of PPC in patients under general anesthesia [17]. MP during OLV and its impact on ventilatory‐associated lung injury and PPCs after thoracoscopic lung resection surgery is still under discussion, and there are yet no recommendations on guidelines or education journals [13]. Liu et al.’s prospective cohort multivariable analysis, including 622 patients, showed no link between MP and PPCs, but their median MP was significantly lower than previous studies (median range from 6.02 to 6.76 J/min) [18]. In our study, patients undergoing uRATS had a MP like Liu et al.’s cohort, lower than the one observed in the mRATS group (median up to 13.8 J/min). More interestingly, in a post hoc analysis of the iPROVE‐OLV RCT with 1.253 patients undergoing thoracic surgery [19], MP greater than 11 J/min was associated with the occurrence of PPC. In the same study, components of MP were assessed separately and found to have different clinical implications. The dynamic resistive MP was associated with worse outcomes in terms of PPC, whereas the static component was found to be protective if related to an individualized PEEP. In our sample, patients in the uRATS without capnothorax pressure applied had PEEP and consequently static MP significantly lower than the mRATS group. Yet, the fact that all our patients had an individualized PEEP applied after a RM might be the reason that they all had their optimal airway opening pressures with the lowest possible elastic and dynamic MP components. As PPCs were not a primary outcome of our study, we lack the sample size and event rate required to establish any relationship between our findings and clinically relevant postoperative outcomes. However, the observed differences in PEEP requirements, airway pressures, MP, and hypercapnia suggest that capnothorax may have a measurable impact on intraoperative respiratory physiology during OLV. Further studies are required to determine whether these physiological differences translate into clinically meaningful outcomes. To date, MP in OLV and interpretation regarding potential clinical benefit should remain appropriately cautious.

Patients in the mRATS group presented significant hypercapnia and a higher RR. Though permissive hypercapnia is advocated during OLV [20], there is little evidence regarding the limit that requires intervention. As a high RR may be a contributor to ventilator‐induced lung injury through the increase of MP, care must be taken not to make the mistake of pursuing a normal CO_2_ value while doing unnecessary harm.

Even if it was not a primary outcome of the study, we found differences in hemodynamic stability and the use of vasopressors with higher noradrenaline dosage in the mRATS group. We hypothesize it is due to the continuous positive intrathoracic pressure of capnothorax in the mRATS group, possibly lowering venous return and cardiac output. These findings warrant further investigation as our experimental protocol did not include advanced hemodynamic monitoring of cardiac output or dynamic preload indices.

There were some minor differences in baseline characteristics between groups, probably due to a limited sample size. The uRATS group had larger FVC when compared to mRATS. Although it may have low clinical relevance, a selection bias cannot be ruled out, and it could influence respiratory mechanics per se. OLV duration also differed between study groups. Although sensitivity analysis including OLV duration as a covariate in the mixed‐effects models did not alter the main findings (Supporting Appendix), it may still have influenced respiratory mechanics, gas exchange and vasopressor use.

Until now, the choice of robotic approach for lung resection has depended primarily on the surgeon, taking into consideration the patient’s anatomy, the location and complexity of the lesion, as well as the operator’s experience. Uniportal and biportal approaches have emerged as feasible alternatives to the conventional multiportal technique, offering potential benefits in terms of reduced invasiveness, postoperative pain, and recovery, while maintaining surgical precision and oncological adequacy [21].

Our study findings suggest that uRATS approaches might be better tolerated, possibly because tension capnothorax has a higher impact on respiratory mechanics and may induce significant hypercapnia and hemodynamic disturbances. Chen et al. propensity score matched comparison of short‐term outcomes after mRATS vs. uRATS [22] on a 353‐patient cohort found less PPC in the uRATS group and believed it was due to lesser injury to the intercostal muscles and the neurovascular bundle confined to one intercostal space, hence with lower postoperative pain and sympathetic overactivation. However, they did not consider the impact of tension capnothorax on respiratory mechanics, which may have contributed to PPC. The selection of the optimal approach should, therefore, be individualized, balancing patient comorbidities, tumor characteristics, and the surgical team’s expertise, thus achieving safe and effective pulmonary resections within the Enhanced Recovery After Surgery era [23]. Therefore, tension capnothorax use should be discussed in a multidisciplinary approach so that patients benefit from a minimally invasive approach without excessive risk.

This study has some limitations. First, it is not a randomized trial. The surgical approach was decided by surgeons, according to their expertise and the patient’s anatomy. Our study conclusions may only generate further hypotheses but are not explanatory. Second, the mRATS group was derived from a historical cohort collected shortly before the prospective uRATS cohort. Although both cohorts were managed at the same institution using the same ventilatory protocol and perioperative management strategy, the use of a historical comparator may still introduce unmeasured confounding factors. Potential differences in case selection, surgical complexity, perioperative management, and team experience and learning curve may have influenced the results. Our findings should be interpreted as physiological and exploratory rather than as evidence of clinical superiority of one robotic approach over another. Fourth, main differences found between study groups in respiratory mechanics may reflect combined effects of surgical approach and capnothorax but study design cannot distinguish the independent contribution of these two factors. Finally, the sample size is small and was designed to describe the main intraoperative respiratory and hemodynamic changes across different robotic approaches. It was not powered to assess postoperative clinical outcomes. Further research is needed to elucidate the differences in outcomes of different techniques.

## 5. Conclusion

Uniportal or biportal robotic‐assisted lung resection without capnothorax is associated with a lower impact on respiratory mechanics than multiportal robotic‐assisted lung resection with tension capnothorax. Further studies are needed to determine whether these findings translate into significant differences in clinical outcomes.

**Nomenclature**
CO_2_
: Carbon dioxide
C_rs_
: Quasistatic respiratory system compliance
DP: Driving pressure
FiO_2_
: Fraction of inspiratory oxygen
mRATS: Multiportal RATS
MP: Mechanical power
OLV: One‐lung ventilation
PEEP: Positive end–expiratory pressure
P_Plat_
: Plateau pressure
PPCs: Postoperative pulmonary complications
RATS: Robotic‐assisted thoracic surgery
RM: Recruitment maneuvers
RR: Respiratory rate
TLV: Two‐lung ventilation
uRATS: Uniportal or biportal RATS
VT: Tidal volume

## Author Contributions

Ricard Navarro‐Ripoll is the first and corresponding author as he designed and led the study. Stefano Italiano, Álvaro Buñuel, David Revuelta, and Manuel López‐Baamonde recruited the patients and their data. Stefano Italiano made the statistical analysis. Ricard Navarro‐Ripoll, Stefano Italiano, Anna Ureña, and Ricard Ramos participated in the first draft of the manuscript. The manuscript was finally written by Ferran Seguí with supervision and corrections from Ricard Navarro‐Ripoll and Carlos Ferrando.

## Funding

No funding was received for conducting this study.

## Disclosure

All authors have read and approved the final version of the manuscript.

## Ethics Statement

This study has been approved by the Ethics Committee of Clinical investigation of Hospital Clinic de Barcelona (HCB/2024/0103), and all participants have signed the informed consent.

## Consent

Please see the Ethics Statement.

## Conflicts of Interest

The authors declare no conflicts of interest.

## Supporting Information

Additional supporting information can be found online in the Supporting Information section.

## Supporting information


**Supporting Information 1** Supporting figure S1: Uniportal, biportal, and multiportal RATS incisions. Supporting figure S2: Experimental protocol. Supporting table S1: Correlation between PEEP and potential covariates after Bonferroni post hoc test. Supporting table S2: Baseline comparison of potential covariates between mRATS and uRATS groups. Supporting table S3: Sensitivity analysis comparing primary and OLV duration‐adjusted linear mixed‐effects models. Supporting table S4: Comparison of respiratory mechanics between mRATS and uRATS after Bonferroni post hoc test (linear mixed‐effects model [LMM]). Supporting figure S3: Comparison of noradrenaline total dose between mRATS and uRATS. Supporting table S5: Comparison of noradrenaline total dose between mRATS and uRATS.

**Supporting Information 2** STROBE Statement—checklist of items that should be included in reports of observational studies.

## Data Availability

Data are provided within the manuscript or supporting information files and are also available from the corresponding author upon reasonable request.
