# Supplementary material for: Impact on Respiratory Mechanics of Uniportal–Biportal Robotic Lung Resection Without Capnothorax Versus Multiportal Approach With Capnothorax: A Prospective Cohort Observational Study
Source: Anesthesiol Res Pract. 2026 Oct 8;2026:5324693. doi: 10.1155/anrp/5324693 (PMC13647211; doi:10.1155/anrp/5324693)
Supplement: Supplementary file 1 [file ANRP-2026-5324693-s002.docx]

**Supplementary Appendix**

Figure S1 Uniportal, biportal and multiportal RATS incisions

Legend of Figure S1: shows from left to right a uniportal approach, a biportal approach and a multiportal approach. With permission from the author: Néstor Quiroga.

Figure S2: Experimental protocol.

Legend Figure S2. T1 is baseline two lung ventilation after intubation. T2 is one lung ventilation (OLV) without recruitment maneuver (RM). T3 is OLV right after RM. T4 is one lung ventilation 20 minutes after the RM. T5 is at the end of one lung ventilation. T6 is at the end of surgery, with two lung ventilation, before extubation. Green boxes between T2-T3 and T5-T6 RM and individualized positive end-expiratory pressure setting. During the entire procedure FiO2 0.8 and respiratory rate (RR) between 15 and 20 breaths per minute were maintained. Checks in blue are respiratory mechanics recordings, and checks in red are arterial blood gas analysis.

**Assessment of Potential Confounding and Model Development**

Prior to specifying the final mixed-effects model, potential confounding variables were identified based on clinical criteria and characteristics of the study design. The model was initially specified for positive end-expiratory pressure (PEEP), which was the primary outcome variable used for sample size estimation. The same modeling structure was subsequently applied to the other dependent variables to ensure methodological consistency. The clinically relevant baseline covariates considered were age and body mass index (BMI).

Confounding was evaluated using a combination of clinical evaluation and statistical analysis.

First, the association between each candidate variable (age and BMI) and PEEP was explored within the URATS group (non-exposed group) using Pearson correlation coefficients. Bonferroni correction was applied to account for repeated measurements across time points. No statistically significant correlations were observed after adjustment.

Table S1: Correlation between PEEP and potential covariates after Bonferroni post-hoc test.

|  |  | **T1** | **T2** | **T3** | **T4** | **T5** | **T6** |
| --- | --- | --- | --- | --- | --- | --- | --- |
| **Age** | r | 0.127 | 0.127 | 0.020 | 0.023 | 0.004 | 0.183 |
| **(years)** | p-value | 1 | 1 | 1 | 1 | 1 | 1 |
| **BMI** | r | 0.380 | 0.380 | 0.370 | 0.406 | 0.390 | 0.212 |
| **(Kg/m2)** | p-value | 0.806 | 0.806 | 0.930 | 0.550 | 0.769 | 1 |

Legend of Table S1. Pearson correlation coefficient (r). PEEP: positive end-expiratory pressure. BMI: body mass index. OLV: One Lung Ventilation

Second, differences in the candidate baseline covariates between mRATS and uRATS groups were assessed using non-parametric tests. No statistically significant between-group differences were observed for age or BMI.

Table S2: Baseline comparison of potential covariates between mRATS and uRATS groups.

|  | **mRATS (n=30)** | **uRATS (n=30)** | p |
| --- | --- | --- | --- |
| Age (years) | 69 (9) | 70.5 (12) | 0.549 * |
| BMI (Kg/m2) | 23 (3.6) | 25.3 (4.7) | 0.304 * |

Legend of Table S2. Data presented as median and IQR. * Wilcoxon (Mann-Whitney) rank-sum test.

**Sensitivity Analysis**

A significant between-group difference was observed for OLV duration (median [IQR], mRATS: 163 [42] min vs uRATS: 113.5 [50] min; p<0.001) and total ventilation time. However, the difference in total ventilation time was primarily attributable to differences in OLV duration, as bipulmonary ventilation time did not differ between groups (39 [27] min vs 46.5 [18] min, respectively; p=0.089). However, it was not included in the primary model because it represents a post-exposure variable, and adjustment for it could potentially influence the estimated effect of the surgical approach.

Considering the clinical relevance of OLV duration to intraoperative ventilatory mechanics, the linear mixed-effects models were repeated with OLV duration included as an additional covariate as a sensitivity analysis.

Table S3: Sensitivity analysis comparing primary and OLV duration-adjusted linear mixed-effects models

| **Outcome** | **Effect** | **Primary model**  **Wald χ²** | **P value** | **OLV-adjusted model**  **Wald χ²** | **P value** |
| --- | --- | --- | --- | --- | --- |
| **PEEP** | Group | 0.03 | 0.858 | 0.51 | 0.475 |
|  | Time | 1693.76 | <0.001 | 1693.86 | <0.001 |
|  | Group × time | 527.41 | **<0.001** | 527.64 | **<0.001** |
| **Plateau pressure** | Group | 0.01 | 0.937 | 0.54 | 0.461 |
|  | Time | 294.06 | <0.001 | 294.07 | <0.001 |
|  | Group × time | 112.92 | **<0.001** | 113.03 | **<0.001** |
| **Driving pressure** | Group | 0.00 | 1.000 | 0.23 | 0.630 |
|  | Time | 102.95 | <0.001 | 102.95 | <0.001 |
|  | Group × time | 10.83 | 0.055 | 10.83 | 0.055 |
| **Compliance** | Group | 0.36 | 0.550 | 0.03 | 0.867 |
|  | Time | 235.69 | <0.001 | 235.67 | <0.001 |
|  | Group × time | 5.50 | 0.358 | 5.50 | 0.358 |
| **Mechanical power** | Group | 0.02 | 0.895 | 0.02 | 0.895 |
|  | Time | 264.96 | <0.001 | 264.96 | <0.001 |
|  | Group × time | 95.37 | **<0.001** | 95.37 | **<0.001** |
| **Static mechanical power** | Group | 0.11 | 0.741 | 0.36 | 0.549 |
|  | Time | 846.32 | <0.001 | 846.38 | <0.001 |
|  | Group × time | 321.47 | **<0.001** | 321.72 | **<0.001** |
| **Respiratory rate** | Group | 0.27 | 0.605 | 0.40 | 0.527 |
|  | Time | 217.11 | <0.001 | 217.11 | <0.001 |
|  | Group × time | 34.49 | **<0.001** | 34.46 | **<0.001** |
| **EtCO2** | Group | 0.09 | 0.770 | 0.01 | 0.913 |
|  | Time | 341.66 | <0.001 | 341.62 | <0.001 |
|  | Group × time | 49.68 | **<0.001** | 49.68 | **<0.001** |

Legend of Table S3: Overall fixed-effect tests derived from linear mixed-effects models including group, time, and the group-by-time interaction as fixed effects, with patient included as a random intercept. Wald χ² are reported. PEEP: positive end-expiratory pressure. EtCO2: end tidal carbon dioxide.

Adjustment for OLV duration did not alter the findings of the primary analysis, with the overall pattern and statistical significance of group, time, and group-by-time effects remaining unchanged across all outcomes.

Table S4: Comparison of respiratory mechanics between mRATS and uRATS after Bonferroni post-hoc test (primary unadjusted linear mixed-effects model (LMM)).

|  | **Statistic** | **T1** | **T2** | **T3** | **T4** | **T5** | **T6** |
| --- | --- | --- | --- | --- | --- | --- | --- |
| **PEEP (cmH2O)** | MD | 0.07 | 0.07 | -5.33 | -5.30 | -5.26 | -0.70 |
|  | (95% CI) | -1.19 to 1.32 | -1.19 to 1.32 | -6.59 to -4.08 | -6.55 to -4.05 | -6.52 to -4.00 | -1.95 to 0.55 |
|  | p value | 1 | 1 | <0.001 | <0.001 | <0.001 | 1 |
| **Plateau pressure (cmH2O)** | MD | 0.07 | -0.70 | -4.20 | -6.53 | -5.60 | -1.20 |
|  | (95% CI) | -2.76 to 2.90 | -3.53 to 2.13 | -7.03 to -1.37 | -9.36 to -3.70 | -8.44 to -2.75 | -4.03 to 1.63 |
|  | p value | 1 | 1 | <0.001 | <0.001 | <0.001 | 1 |
| **Driving pressure (cmH2O)** | MD | 0.00 | -0.93 | 1.20 | -1.23 | -0.33 | -0.50 |
|  | (95% CI) | -2.47 to 2.47 | -3.41 to 1.54 | -1.27 to 3.67 | -3.71 to 1.24 | -2.82 to 2.16 | -2.97 to 1.97 |
|  | p value | 1 | 1 | 1 | 1 | 1 | 1 |
| **Compliance (ml/cmH2O)** | MD | -1.74 | 1.16 | -2.82 | 3.42 | 0.69 | 1.20 |
|  | (95% CI) | -11.53 to 8.05 | -8.64 to 10.95 | -12.61 to 6.97 | -6.37 to 13.21 | -9.16 to 10.53 | -8.65 to 11.04 |
|  | p value | 1 | 1 | 1 | 1 | 1 | 1 |
| **Mechanical power (J/min)** | MD | -0.95 | -0.26 | -2.16 | -4.80 | -4.98 | -2.37 |
|  | (95% CI) | -3.33 to 1.44 | -2.65 to 2.13 | -4.55 to 0.24 | -7.19 to -2.41 | -7.38 to -2.58 | -4.76 to 0.02 |
|  | p value | 1 | 1 | 0.163 | <0.001 | <0.001 | 0.055 |
| **Static (J/min)** | MD | -0.11 | -0.02 | -2.56 | -3.36 | -3.65 | -0.96 |
|  | (95% CI) | -1.20 to 0.99 | -1.11 to 1.08 | -3.66 to -1.47 | -4.45 to -2.27 | -4.74 to -2.55 | -2.06 to 0.13 |
|  | p value | 1 | 1 | <0.001 | <0.001 | <0.001 | 0.197 |
| **Respiratory Rate (breath/min)** | MD | -0.17 | -0.07 | 0.47 | -1.30 | -1.41 | -0.67 |
|  | (95% CI) | -1.25 to 0.92 | -1.15 to 1.02 | -0.62 to 1.55 | -2.39 to -0.21 | -2.51 to -0.32 | -1.75 to 0.42 |
|  | p value | 1 | 1 | 1 | 0.004 | 0.001 | 1 |
| **EtCO2 (mmHg)** | MD | 0.37 | -0.07 | -5.67 | -5.37 | -3.36 | 0.02 |
|  | (95% CI) | -3.86 to 4.60 | -4.30 to 4.16 | -9.90 to -1.44 | -9.60 to -1.14 | -7.64 to 0.91 | -4.24 to 4.27 |
|  | p value | 1 | 1 | <0.001 | 0.001 | 0.529 | 1 |

Legend Table S4. Mean difference (MD) and 95% Confidence Interval (CI) between mRATS and uRATS groups. PEEP: positive end-expiratory pressure. EtCO_2_: end tidal carbon dioxide.

Figure S3: Comparision of noradrenaline total dose between mRATS and uRATS.

Legend of Figure S3. Data presented as median and IQR. * Wilcoxon (Mann-Whitney) rank-sum test.

Table S5: Comparison of noradrenaline total dose between mRATS and uRATS.

|  | **MRATS (n=30)** | **URATS (n=30)** | **P value** |
| --- | --- | --- | --- |
| **Noradrenaline total dose (μg)** | 352 (400) | 136 (372) | **0.006 *** |

Legend of Table S5. Data presented as median and IQR. * Wilcoxon (Mann-Whitney) rank-sum test.
